# A Linnaeus NG ^TM^ interactive key to the Lithocolletinae of North-West Europe aimed at accelerating the accumulation of reliable biodiversity data (Lepidoptera, Gracillariidae)

**DOI:** 10.3897/zookeys.422.7446

**Published:** 2014-07-03

**Authors:** Camiel Doorenweerd, Merel M. van Haren, Maarten Schermer, Sander Pieterse, Erik J. van Nieukerken

**Affiliations:** 1 Naturalis Biodiversity Center, Department of Terrestrial Zoology, P.O. Box 9517, 2300 RA, Leiden, the Netherlands; 2 Naturalis Biodiversity Center, ETI BioInformatics, P.O. Box 9517, 2300 RA, Leiden, the Netherlands; 3 Naturalis Biodiversity Center, Educational Development, P.O. Box 9517, 2300 RA, Leiden, the Netherlands; 4 Radboud University, RU-Institute for Water and Wetland research, Department of Animal ecology and ecophysiology, P.O. Box 9010, 6500 GL, Nijmegen, the Netherlands

**Keywords:** *Cameraria*, *Phyllonorycter*, *Macrosaccus*, *Triberta*, identification, monitoring, conservation, biodiversity, leafminers

## Abstract

We present an interactive key that is available online through any web browser without the need to install any additional software, making it an easily accessible tool for the larger public. The key can be found at http://identify.naturalis.nl/lithocolletinae. The key includes all 86 North-West European Lithocolletinae, a subfamily of smaller moths (“micro-moths”) that is commonly not treated in field guides. The user can input data on several external morphological character systems in addition to distribution, host plant and even characteristics of the larval feeding traces to reach an identification. We expect that this will enable more people to contribute with reliable observation data on this group of moths and alleviate the workload of taxonomic specialists, allowing them to focus on other new keys or taxonomic work.

## Introduction

Taxonomic identification is the key to aggregating knowledge about species. We are increasingly aware that we live in a changing world and that different species respond differently to changes (Bellard et al. 2012; Butchart et al. 2010; Fernandez-Triana et al. 2011; Heller and Zavaleta 2009; Pereira et al. 2010; Thomas et al. 2004). Statistical models that elucidate the impact of environmental change on ecological communities in future scenarios depend on appropriate underlying faunistic data (Ellis et al. 2013; Soberon and Peterson 2009; ter Steege et al. 2013). Current biodiversity indicators that have potential to be included in such models often rely on monitoring data of relatively restricted systematic groups. Commonly these are aesthetically attractive, such as birds (Schmeller et al. 2012), butterflies (Dover et al. 2011) and dragonflies (Parr 2010) or have a societal impact such as bees (Schindler et al. 2013). For these groups momentum can be generated amongst the general public to gather and contribute faunistic data to large databases. There are, however, new opportunities arising to expand to other groups of organisms that may strengthen biodiversity models. The accessibility of information as well as possibilities to submit data through the internet enables a new generation of observers to contribute to databases, such as www.observado.org or www.lepiforum.de. A genuine concern with this method of data collection is the reliability of the identifications. Although most databases strongly suggest including photographs with each observation, this recommendation is not always followed, and may not even be feasible with, for instance, trap samples consisting of numerous specimens. Furthermore, even when a photograph is present it may not show the relevant characters or there are simply not enough taxonomic specialists available to verify all sightings. Instead of using an approach of ‘damage control’ by attempting to verify all sightings, it may therefore be more efficient for taxonomic specialists to provide better *a priori* information through identification tools that are tailored to these growing groups of enthusiasts.

For the past two centuries identifications of organisms have usually been carried out with dichotomous keys. One of the governing issues with dichotomous keys is that they “are compiled by those who do not need them for those who cannot use them” (http://www.zin.ru/Animalia/Coleoptera/eng/syst8.htm). Although useful dichotomous keys certainly exist, they always have the disadvantage of being static and cannot be adjusted easily with new taxonomic insights. Furthermore, it is impossible to skip certain couplets when a required character is missing or not visible. Often, several keys are needed for a single group to target different developmental stages, sexes or character sets (e.g. external characters, genitalia). The total amount of couplets in a key is the amount of taxa included, minus one. The more questions a key contains, the less likely there will be an accurate identification (Osborne 1963). As the taxa in a key are ‘fixed’, a species not included in it may nevertheless easily end up in a couplet, thus giving a wrong identification. Different alternatives have been proposed for dichotomous keys, with more recently computer aided ones, usually called ‘interactive keys’ (Penev et al. 2012). Although a review of all present methods is beyond this paper (but see Dallwitz 2000; Dallwitz et al. 2000 onwards; Farr 2006; Walter and Winterton 2007), the vast majority of them have issues which make them unsuitable for a larger public. These issues include the necessity to install and possibly purchase software, which may or may not be available for all operating systems, and the need for training due to the complexities of the particular software package.

Butterflies are often used as biodiversity indicators, but only represent a small proportion of the order Lepidoptera (Mutanen et al. 2010; van Nieukerken et al. 2011; Regier et al. 2013). Most Lepidoptera are herbivores of higher plants with different degrees of specialization (Menken et al. 2010; Powell et al. 1998). Relationships with higher trophic levels include a wide variety of parasitoids and insectivores. These insectivores include mammals with protected conservation status, such as some species of bats. The diversity of Lepidoptera in an area thus relays information about a whole ecosystem. Among enthusiasts that go night-collecting to observe moths there is a tendency to only identify species of a selection of families that mainly contain larger moths (“macro-moths”) which have traditionally been treated in field guides. For the interactive key presented here we therefore selected the subfamily Lithocolletinae in a family with smaller moths (Gracillariidae), limiting ourselves for practical reasons to all 86 species from North-West Europe (Buszko 2013) (see Table 1 for names and authorities). Lithocolletinae in Europe are predominantly represented by the species rich genus *Phyllonorycter* Hübner, 1822, with 128 known species (Buszko 2013). The subfamily is further represented by three genera each with one species in the area: *Cameraria* Chapman, 1902 with the well-known horse-chestnut pest *Cameraria ohridella*, and the recently established genera *Macrosaccus* Davis & De Prins, 2011 and *Triberta* De Prins, 2013 (see Davis and De Prins 2011, De Prins et al. 2013). All species within the subfamily have comparable wing pattern elements and are similar in size and morphology. This makes them superficially very similar, but most species can be identified using a combination of several wing pattern elements. Furthermore, searching for larval feeding traces on plants is an effective alternative to collecting adults for gathering faunistic data. The majority of Lithocolletinae larvae feed inside leaves, where they consume parenchyma but leave the epidermis intact. This results in damage that is usually referred to as a leafmine. The high degree of monophagy (Lopez-Vaamonde et al. 2003) allows for a reliable identification in most cases through a combination of an identified host plant and several characters of the larval feeding method.

**Table 1.** The 86 species included in the key in alphabetical order.

*Cameraria ohridella* Deschka & Dimić, 1986	*Phyllonorycter lantanella* (Schrank, 1802)
*Macrosaccus robiniella* (Clemens, 1859)	*Phyllonorycter lautella* (Zeller, 1846)
*Phyllonorycter abrasella* (Zeller, 1846)	*Phyllonorycter leucographella* (Zeller, 1850)
*Phyllonorycter acaciella* (Duponchel, 1843)	*Phyllonorycter maestingella* (Müller, 1764)
*Phyllonorycter acerifoliella* (Zeller, 1839)	*Phyllonorycter mannii* (Zeller, 1846)
*Phyllonorycter aemula* Triberti, Deschka & Huemer, 1997	*Phyllonorycter medicaginella* (Gerasimov, 1930)
*Phyllonorycter agilella* (Zeller, 1846)	*Phyllonorycter mespilella* (Hübner, 1805)
*Phyllonorycter alpina* (Frey, 1856)	*Phyllonorycter messaniella* (Zeller, 1846)
*Phyllonorycter anderidae* (W. Fletcher, 1885)	*Phyllonorycter millierella* (Staudinger, 1871)
*Phyllonorycter apparella* (Herrich-Schäffer, 1855)	*Phyllonorycter monspessulanella* (Fuchs, 1897)
*Phyllonorycter blancardella* (Fabricius, 1781)	*Phyllonorycter muelleriella* (Zeller, 1839)
*Phyllonorycter brevilineatella* (Benander, 1944)	*Phyllonorycter nicellii* (Stainton, 1851)
*Phyllonorycter cavella* (Zeller, 1846)	*Phyllonorycter nigrescentella* (Logan, 1851)
*Phyllonorycter cerasicolella* (Herrich-Schäffer, 1855)	*Phyllonorycter oxyacanthae* (Frey, 1855)
*Phyllonorycter cerasinella* (Reutti, 1853)	*Phyllonorycter parisiella* (Wocke, 1848)
*Phyllonorycter comparella* (Duponchel, 1843)	*Phyllonorycter pastorella* (Zeller, 1846)
*Phyllonorycter connexella* (Zeller, 1846)	*Phyllonorycter platani* (Staudinger, 1870)
*Phyllonorycter coryli* (Nicelli, 1851)	*Phyllonorycter populifoliella* (Treitschke, 1833)
*Phyllonorycter corylifoliella* (Hübner, 1796)	*Phyllonorycter quercifoliella* (Zeller, 1839)
*Phyllonorycter cydoniella* (Denis & Schiffermüller, 1775)	*Phyllonorycter quinqueguttella* (Stainton, 1851)
*Phyllonorycter delitella* (Duponchel, 1843)	*Phyllonorycter rajella* (Linnaeus, 1758)
*Phyllonorycter deschkai* Triberti, 2007	*Phyllonorycter roboris* (Zeller, 1839)
*Phyllonorycter distentella* (Zeller, 1846)	*Phyllonorycter rolandi* (Svensson, 1966)
*Phyllonorycter dubitella* (Herrich-Schäffer, 1855)	*Phyllonorycter sagitella* (Bjerkander, 1790)
*Phyllonorycter emberizaepenela* (Bouché, 1834)	*Phyllonorycter salicicolella* (Sircom, 1848)
*Phyllonorycter esperella* (Goeze, 1783)	*Phyllonorycter salictella* (Zeller, 1846)
*Phyllonorycter eugregori* Laštůvka & Laštůvka, 2006	*Phyllonorycter scabiosella* (Douglas, 1853)
*Phyllonorycter fraxinella* (Zeller, 1846)	*Phyllonorycter schreberella* (Fabricius, 1781)
*Phyllonorycter froelichiella* (Herrich-Schäffer, 1855)	*Phyllonorycter scitulella* (Duponchel, 1843)
*Phyllonorycter geniculella* (Ragonot, 1874)	*Phyllonorycter scopariella* (Zeller, 1846)
*Phyllonorycter gerasimowi* (Hering, 1930)	*Phyllonorycter sorbi* (Frey, 1855)
*Phyllonorycter harrisella* (Linnaeus, 1761)	*Phyllonorycter spinicolella* (Zeller, 1846)
*Phyllonorycter heegeriella* (Zeller, 1846)	*Phyllonorycter staintoniella* (Nicelli, 1853)
*Phyllonorycter heringiella* (Grønlien, 1932)	*Phyllonorycter stettinensis* (Nicelli, 1852)
*Phyllonorycter hilarella* (Zetterstedt, 1839)	*Phyllonorycter strigulatella* (Zeller, 1846)
*Phyllonorycter hostis* Triberti, 2007	*Phyllonorycter tenerella* (de Joannis, 1915)
*Phyllonorycter ilicifoliella* (Duponchel, 1843)	*Phyllonorycter trifasciella* (Haworth, 1828)
*Phyllonorycter insignitella* (Zeller, 1846)	*Phyllonorycter trifoliella* (Gerasimov, 1933)
*Phyllonorycter issikii* (Kumata, 1963)	*Phyllonorycter tristrigella* (Haworth, 1828)
*Phyllonorycter joannisi* (Le Marchand, 1936)	*Phyllonorycter ulicicolella* (Stainton, 1851)
*Phyllonorycter junoniella* (Zeller, 1846)	*Phyllonorycter ulmifoliella* (Hübner, 1817)
*Phyllonorycter klemannella* (Fabricius, 1781)	*Phyllonorycter viminetorum* (Stainton, 1854)
*Phyllonorycter kuhlweiniella* (Zeller, 1839)	*Triberta helianthemella* (Herrich-Schäffer, 1860)

Keys that currently exist for Lithocolletinae or *Phyllonorycter* treat restricted geographic regions and are mostly for adults only. Examples include a key for the British Isles (Emmet et al. 1985), for France and the British isles (Bradley et al. 1969) and for Fennoscandia and Denmark (Bengtsson and Johansson 2011). Specifically for the larval feeding traces on plants there is a dichotomous web-key covering the whole of Europe (Ellis 2014). For the British Isles there is also an interactive key available for the genus *Phyllonorycter*, using the Delta-Intkey platform (Watson and Dallwitz 2003 onwards). To address the need for accessible identification methods targeting groups of animals that have potential to be important for biodiversity estimates, we present in this paper an initial step in this direction through a key using the Linnaeus NG platform that includes all North-West European species of Lithocolletinae.

This paper is formatted following the guidelines for interactive keys (Penev et al. 2012).

## Software technical specification

Linnaeus NG is a web-based species information management system. Linnaeus NG comprises several modules, such as a species description module, a module for plotting distribution, and two types of keys. For this study, the multi-entry key was employed. The data underlying the key are managed in a spreadsheet and can be uploaded to a Linnaeus NG project as comma separated value (.CSV) files by project administrators. Two different files need to be uploaded. One contains a matrix with species data, characters, states and the relation between species and states. A second file contains image links for all character states. Alternatively, these values can be added and edited directly through the web-based multi-entry key management interface. This interface also contains an upload facility for supplying images for states and, optionally, species. Linnaeus NG is developed using open source techniques (PHP, MySQL) and is hosted in a Linux environment. On the client-side, project administrators interact with the program solely through a web browser. Recent versions of all major browsers are supported, for regular platforms and tablets. Currently, Linnaeus NG is proprietary software; updates and changes can only be made in agreement with Naturalis Biodiversity Center. However, access to Linnaeus NG is not limited to employees or associates of Naturalis Biodiversity Center and can be granted on request.

### User interface

Users can access the key at: http://identify.naturalis.nl/lithocolletinae and fully use the key online through any web browser. No additional software is required. The interface was designed to be intuitive and graphic, using detail images to explain different character states and directly showing the effects of each choice by only displaying images of the remaining possible outcomes. This combination of character state selection and general visual recognition of candidate species prevents users from having to select character states until only a single option remains.

**Figure 1.**
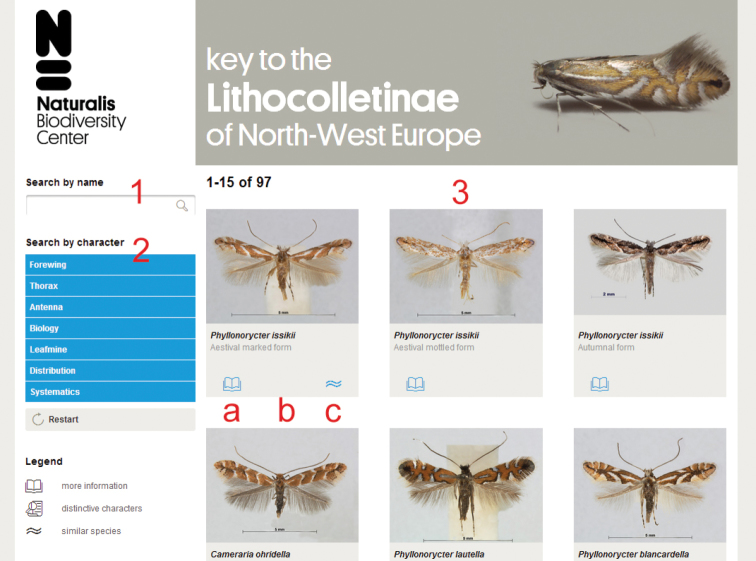
Screenshot of the user interface with different sections indicated. **1** Search by name **2** search by character **3** main window with resulting selection, **a** more information, links to respective species page on fauna europaea **b** distinctive characters, becomes visible with a selection of 8 or less **c** displays species with a similar appearance.

Each possible outcome of the key is represented by a thumbnail photograph of a mounted adult specimen in the main section (Figure 1: 3). Photographs were taken using stacking photography with a motorized Zeiss V20 with MRc5 camera and Axiovision software. During post-processing, photographs were sharpened, reduced in size to 800×600 pixels and backgrounds were homogenized in Adobe Photoshop CS5®. The main section of the key starts with all 96 possible outcomes, of which only the first 15 will be shown initially to prevent long loading times. Scrolling down and clicking the “show more results” button will show more options with increments of 15. Photographs can be enlarged by clicking on the thumbnail. By using the forward and previous buttons in the image overlay, the user can navigate through all remaining search results. There are three buttons below each thumbnail. The left symbol (Figure 1: a) provides additional information through an external link to the corresponding species page on Lepiforum.de. The centre symbol (Figure 1: b) is activated when less than eight options remain and lists the differences in character states. The symbol on the right (Figure 1: c) shows which outcomes are highly similar, when applicable. The selection of highly likely outcomes is based on indications in literature or personal experience of the authors, there is no automated algorithm involved.

A user can directly search for a species they suspect they might be trying to identify by using the search box (Figure 1: 1). Alternatively, identification may be reached by selecting character states. The section beneath the search option (Figure 1: 2) lists the character groups, which will expand upon clicking and show all characters within that group. As with any interactive key the user is free to choose which character to begin with. There are three morphological character groups for adult specimens: forewing, thorax and antenna. Of these, only the forewing is further subdivided, into 11 characters. Once a character has been selected, a pop-up will appear on the screen with a brief description and 200×150 pixel detail images representing the different character states. After making a choice the resulting selection is shown in the main section (Figure 1: 3). Instead of just focussing on adults, the key can also be used to identify larvae or leafmines. The characters in the sections biology, distribution and systematics are not morphological, but may help with the identification nonetheless. Alternatively, these options may be used to quickly get a graphic overview of a given species group in a certain country, or all species found on a particular family of host plants.

### Taxonomic coverage

There are 97 possible outcomes of the key, representing 86 species (Table 1). The majority of outcomes coincide with species, but some species that exhibit intraspecific variation are subdivided. There are species with clearly different forms, such as the aestival and autumnal form of *Phyllonorycter issikii* (Huisman et al. 2013) or the *irmella* “form” of *Phyllonorycter lautella*, which was at one point regarded a different species (van Nieukerken et al. 2001). In other cases it is less obvious, with a darker and lighter form, sexual dimorphism or the variable presence or absence of a fascia. Because highly variable forms will persist in any selection and hamper an efficient identification, it has often been decided to split these into different outcomes. A positive side effect of this is that the images of several forms give the user some indication on the intraspecific variation.

The 86 species (Table 1) that are included make the key complete for all species found in North-West Europe, more specifically those reported as present in Fauna Europaea (Buszko 2013) in: Austria, Belgium, Czech Republic, Denmark, Finland, Germany, Great Britain, Ireland, Luxembourg, The Netherlands, Norway, Poland, Slovakia, Sweden and Switzerland. In practice, the key will also be functional in the northern half of France. Although sometimes disputed (Baryshnikova 2012), we treat *Phyllonorycter distentella* and *Phyllonorycter mannii* as distinct species based on a difference in wing pattern and DNA barcode data (Lopez-Vaamonde et al. in prep.). On the other hand, there are no clear morphological, life history or molecular characters to support a split between *Phyllonorycter pyrifoliella* and *Phyllonorycter gerasimowi*. We therefore treat *Phyllonorycter pyrifoliella* as a junior synonym (Zdeněk Laštůvka, pers. comm.).

### Metadata

The selection of morphological characters (Table 2) was initially based on existing dichotomous keys and we follow the terminology used therein. The white ground colour of the forewings for example is the starting point in both the NationalNyckeln (Bengtsson and Johansson 2011) and Bradley’s key (1969). Only characters that are applicable to virtually all taxa were included. All previously used characters mainly relied on forewing pattern. We added several characters that can be found on the head, thorax and antennae of adults. After beta-testing a preliminary version of the key with a varied group of novice and advanced identifiers, we found that several characters were unsuitable. The main character leading to faulty identifications was the colour of the head tuft. Judging a colour proved too subjective in general, especially when identifying from photographs. Moreover, the character was variable in many species. Head tuft colour was therefore removed from the key. The forewing ground colour is traditionally vividly described in the literature, with states including shining ochreous, pale golden brown, shining pale golden ochreous, dark brown to golden brown etc. (Emmet et al. 1985). Because of the subjective nature and dependence on lighting conditions, we opted for a simplified colour scheme of ‘white’ and ‘other’ for this key.

**Table 2.** Morphology characters and states.

Character group	Character	States
Forewing	Forewing ground colour	White; other
Forewing	Forewing mottling	Mottled; no mottling
Forewing	Forewing basal streak	Present; absent
Forewing	Forewing basal streak contour	None; costal; bilateral
Forewing	Forewing fascia	0; 1; 2; 3
Forewing	Forewing costals	0; 1; 2; 3; 4
Forewing	Forewing dorsals	0; 1; 2; 3; 4
Forewing	Forewing markings contour	Unilateral basal; unilateral proximal; bilateral; absent
Forewing	Forewing apical marking	Dot; stripe; mottled; absent
Forewing	Forewing cilia line	Present; absent
Forewing	Forewing apical fringe	With markings; uninterrupted
Thorax	Thorax pattern	Striped; uniform; silver or golden
Antenna	Antenna colour pattern	Even or chequered; black tip; white tip
Leafmine	Leafmine orientation	Tentiform underside; Tentiform upperside; Full depth blotch; Epidermal upperside blotch; Stem-mine
Leafmine	Leafmine location	Along secondary veins; Along main vein; Leaf base; Leaf margin; Leaf lobe; Whole leaf; Stem; Rachis wings
Leafmine	Leafmine ribs	None; 1; Several; Many
Leafmine	Leafmine frass	Linear; Aggregated; Scattered; Attached to cocoon

Lithocolletinae are all herbivores in their larval life stage with commonly a high degree of monophagy. The host plant is therefore often important for the identification. The key includes data on: host family, host genus and host genus/species (Table 3). Host genus and host genus/species partly overlap. The list ‘host genus/species’ includes all 303 known host species for the 86 Lithocolletinae (De Prins and De Prins 2014), but also includes records where there is only a generic identification of the host. Generic host associations are repeated in the separate list ‘host genus’ because this results in a significantly shorter list of options and may be preferred by the user. Characters of the larva and feeding method are included under “Leafmine” (Table 2). Early larval instars are sap-feeding only, but during later instars plant tissue is consumed and excreted in small pellets called “frass”. The positioning of the frass in the mine is often diagnostic. The relative place and orientation of the mine on the leaf is also of importance. Mines can be “full depth”, where everything except the two epidermal layers is eaten, or “epidermal” at the upper or lower side of the leaf, where the parenchyma is partly left intact. The epidermis of either mine type may contain ribs. The mines’ position on the leaf, for example along a vein or in a leaf lobe, is often diagnostic and can be helpful with plants that are host to many species of Lithocolletinae. Further information that can be valuable for narrowing the selection is distribution information and the taxonomic species group. All character states in Table 2 are represented by images, the different states for the characters in Table 3 are presented in an alphabetical list. All lists can be searched by using the browsers’ search function, commonly accessed through CTRL+F (Windows) or Command+F (Apple).

**Table 3.** Non-morphological characters.

Character group	Character
Biology	Host family
Biology	Host genus
Biology	Host genus/species
Distribution	Country
Systematics	Species group

## Discussion

The key presented here for the Lithocolletinae of North-West Europe includes more species, covers a larger area and can be used for more life stages than any existing key for this subfamily. As such, it enables a large potential user group to identify Lithocolletinae through different approaches with minimized specialist effort involved, including future effort regarding updates. However, accomplishing this has not been without challenges. Most existing keys treat the species of a relatively restricted region, often a single country. A larger area holds more species and thus more candidate species that have to be ruled out. This can be circumvented by first selecting a country under distribution. However, inherent to an interactive key, the user has to make this choice actively. On the other hand, having more species in the key than just those already known and published for a country may enable recognition of introduced, migrating or previously overlooked species. A second challenge posed by covering North-West Europe involves the differences in voltinism at different altitudes. Species that are strictly univoltine in northern Sweden may be bivoltine in a warmer climate in Belgium. Adult flight period(s) or larval feeding period(s) could therefore not be included in our key. An advantage that dichotomous keys have over interactive keys in general is that they can include characters that are specific for a selection of taxa. An interactive key is based on a character matrix where for each taxon all character states need to be filled out; in a dichotomous key, there may be certain species pairs or groups that have distinguishing characters that are lacking in all other taxa (e.g. Laštůvka et al. 2013; Triberti 2007). In other cases, neither external adult characters, larval feeding characters, nor distribution data is sufficient to separate similar species and genitalia need to be examined. Genitalia characters are not included in the key because they are difficult to describe in a quantitative manner. There are plans to include an option in a future version of the key to switch from images of mounted adults to views of different stages or parts, such as the leafmines or genitalia, but this has not yet been implemented.

Aside from the challenges, this key also contains many advantages over existing keys. Perhaps one of the most crucial is the ability to combine quantitative characters with the human brain’s ability to recognize subjective visual patterns (DiCarlo et al. 2012). In a dichotomous key, there is little indication of the remaining taxa and a user can only hope that the final outcome, usually just a species name, matches the candidate. Taxonomic specialists often rely on their extensive exposure to species for identifying them just as much as memorizing the distinguishing characters. The general impression of size and shape can be used in our key as a leading method for the identification by browsing images, or combined with selecting character options and visually verifying that the selection still might contain the species that is attempted to be identified. Optimally, a fast and reliable identification is reached by selecting several characters and visually selecting the best candidate from the remaining selection. Pre-release testing on photographs of live specimens from a regional observers databases by a group composed of novice and experienced identifiers (in a fashion that did not allow for statistical testing) indicated that an identification can be reached for most specimens by selecting three or four states only, with up to nine states for taxonomically difficult species or worn specimens. Over time, a user will become more experienced and will know which characters will be most useful when using the key.

Data on the larval life stage, host plant and distribution were included to take further advantage of all the benefits an interactive key has to offer. Several cautionary notes need to be taken into consideration with these characters. The list of host species is not cross-referenced with distribution. For example, a species may be recorded to feed on *Acer pseudoplatanus* in Germany, but not in Great Britain. If a user in Great Britain thus selects *Acer pseudoplatanus* as host species and Great Britain as country, they may end up with a selection of Lithocolletinae that includes false positives. On the other hand it can broaden the view of the user and allow for earlier recognition of new host records for a country, similar to how new species records for a country can be enabled by not narrowing down to a country first. When using the key, it should be advised to consult regional literature or websites on the resulting identification to see if this may be the case. Using a combination of several fairly easy characters of the larval feeding traces and an identified host should in most cases provide a reliable identification. The key can thus be used to record Lithocolletinae not only during their flight period, but also during the larval feeding period and greatly expand on the amount of faunistic data.

The target audience for this key is limited by the requirement that the insect first has to be recognised or identified as belonging to the Lithocolletinae. However, Lithocolletinae is a species rich subfamily with between 33 (Luxembourg) and 84 species (Austria) per country with distinct adult and larval features that separate them from other Lepidoptera. This makes them generally recognizable by professional lepidopterists and enthusiasts alike. The connection of the 86 Lithocolletinae in this key with 303 host plant species further indicates that the subfamily is an important component of most ecosystems in North-West Europe. Collecting faunistic data on Lithocolletinae has potential to contribute to biodiversity studies, and hopefully more interactive keys with this objective for other Lepidoptera groups will be created by taxonomic specialists to enable more enthusiasts to contribute their data to databases.

## Conclusion

We expect that the key presented here for Lithocolletinae of North-West Europe enables more people to contribute faunistic data with reliable identifications. The key has been designed to allow easy access for inexperienced users, yet still be an efficient tool for advanced users. This publication marks the release of version 1.0. Future changes will be noted under the version history at the website. We will greatly appreciate feedback from users and we hope to further expand and improve the key. Ultimately, we hope to include all European Lithocolletinae, and develop databases with reliable faunistic knowledge that can be useful for biodiversity estimates.
